# A concept analysis of fear of falling in older adults: insights from qualitative research studies

**DOI:** 10.1186/s12877-023-04364-5

**Published:** 2023-10-11

**Authors:** Dayeon Lee, Sunghee H Tak

**Affiliations:** 1 College of Nursing, Seoul National University, Seoul, Republic of Korea; 2 The Research Institute of Nursing Science, College of Nursing, Seoul National University, Seoul, Republic of Korea

**Keywords:** Concept Analysis, Fear of falling, Older adults, Nursing

## Abstract

**Background:**

Fear of falling is a persistent concern about falls that commonly occur in older adults. Recently, it has been argued that fear of falling doesn’t simply mean a state of low falls efficacy, but is a concept distinct from falls efficacy. However, the two concepts are still indistinguishable. Therefore, it is necessary to understand the unique characteristics of the fear of falling. This study aims to analyze the concept of ‘fear of falling’ faced by older adults.

**Methods:**

This study is designed as a concept analysis. A concept analysis was conducted by Walker & Avant’s eight-step concept analysis method. A total of 16 pieces of literature were selected by inclusion and exclusion criteria from those published in Pubmed and Scopus between 1993 and 2022 on 8 November 2022.

**Results:**

Two antecedents, four attributes, and five consequences were identified. Apprehension caused by the unpredictable nature of falls, unease related to one’s vulnerability, high vigilance-related to the environment, and concern about potential harm after fall events were presented as attributes of fear of falling in older adults. There were two antecedents of fear of falling which were awareness of falls and near falls, and direct/indirect experience about falls and near falls. As consequences of fear of falling, protective effect, activities curtailment, reduction in radius of living, restricted freedom, and limited social activities were reported.

**Conclusion:**

It was confirmed that falls and the fear-inducing process were fused to constitute the unique characteristics of the fear of falling. This can be presented as an important basis for future research on the fear of falling or dealing with various aspects of the fear of falling in the clinical field.

## Summary statement

There has been confusion in defining and using the concept of fear of falling. This study clarifies the unique attributes, antecedents, and consequences of fear of falling.

This will establish the concept of fear of falling in future studies and will serve as the basis for dealing with various attributes of fear of falling in the clinical field.

## No patient of public contribution

The articles used for analysis were searched through several search engines, and there was no direct contact with patients or general older adults during the entire study. Since the analysis was conducted based on the contents of the published articles, there is no direct contribution from various users, however since these documents were based on interviews with various patients or older adults, it can be seen that there is an indirect contribution.

## Introduction

The fear of falling is a common problem for older adults. The persistent concern about falls has the potential to have serious consequences, making older adults avoid activities they can perform [1–3]. The fear of falling occurs in 20–85% of older adults, of which 20–55% experience restrictions on daily activities. About 30–50% of older adults, who live independently, experience fear of falling regardless of whether they fall or not [2, 4]. In a previous study, fear of falling was defined as persistent concern about falls that prevent individuals from performing activities they are capable of [5], which meant having low self-efficacy to avoid falls during harmless activities that were essential for daily life [1].

In particular, fear of falling is related to falls and gait disorders [3, 6]. It was also suggested that excessive fear of falling can lead to decreased confidence, activity, and muscle strength, and can lead to decreased physical function [2, 3, 7]. A previous study showed that older adults who experience fear of falling were two to seven times more likely to exhibit decreased physical function and showed a higher dependence on ADL and IADL activities such as walking outside than those who didn’t [8]. In another previous study, it was reported that the fear of falling was associated with activity avoidance, decreased independence, diminished confidence, depression, and a lower quality of life. As a result of the fear of falling, older adults experienced physical, social, and psychological impacts [9].

As such, there is a correlation between fall and fear of falling, and in some previous studies, both of them reported as important predictors of each other. However, there is inconsistent evidence regarding which one causes the other [10–14]. Fear of falling is often used interchangeably with the concepts of falls efficacy, balance confidence, and gait confidence [15]. For example, it is unclear what unique attributes distinguish fear of falling from falls efficacy. Due to this ambiguity, each concept was used interchangeably in previous studies, and similar scales were used to measure each concept [15]. The use of the scales developed to measure other concepts to measure fear of falling is related to the underlying theory that assesses the process of perceived fear of falling. For example, Tinetti et al. [16] developed Falls Efficacy Scale (FES) after defining fear of falling as a state of low falls efficacy based on Bandura’s self-efficacy theory [10, 16]. Recently, it has been argued that fear of falling doesn’t simply mean a state of low falls efficacy, but that fear of falling and falls efficacy are distinct concepts [10, 17]. Evidence suggests that falls efficacy acts as a mediator between fear of falling and falls [10]. Therefore, before understanding and intervening fear of falling in older adults, it is necessary to understand the unique characteristic of the fear of falling which distinguishes it from other concepts. Thus, this study aims to identify the attributes, antecedents, and consequences of older adults’ fear of falling, based on the contents of previous studies.

## Methods

### Data sources

Documents related to the keywords below, published between 1993 and 2022, were searched in Pubmed and Scopus on 8 November 2022. The keywords employed were (“Fear of falling” or “FOF,” “Post-fall syndrome” or “Post fall,” “Anxiety,” “Fear”) and “Fall” and (“Older*,” “Elder*,” or “Geriatric*”) and (“Qualitative” or “Interview”). To provide specific and detailed explanations of the concept, the search was limited to qualitative studies or studies containing interviews. As a result, 93 documents from Pubmed, and 363 documents from Scopus were searched. Among them, 77 documents were removed because of duplication, and some of the remaining 379 documents were removed after checking the title and manuscript. After that, two documents were added through a manual search.

### Data selection

The inclusion criteria are as follows. (1) A study described in Korean or English and accessible as the full text. (2) A study was conducted to identify the concept, characteristics, or attributes of fear of falling.

The exclusion criteria are as follows. (1) A study that uses fear of falling only as a variable to describe a specific object, variable, or program and doesn’t explain related concepts or characteristics. (2) A study that doesn’t have a clear conceptual definition, referring only to repetitive concepts or scale identification and evaluation.

**Fig. 1:**
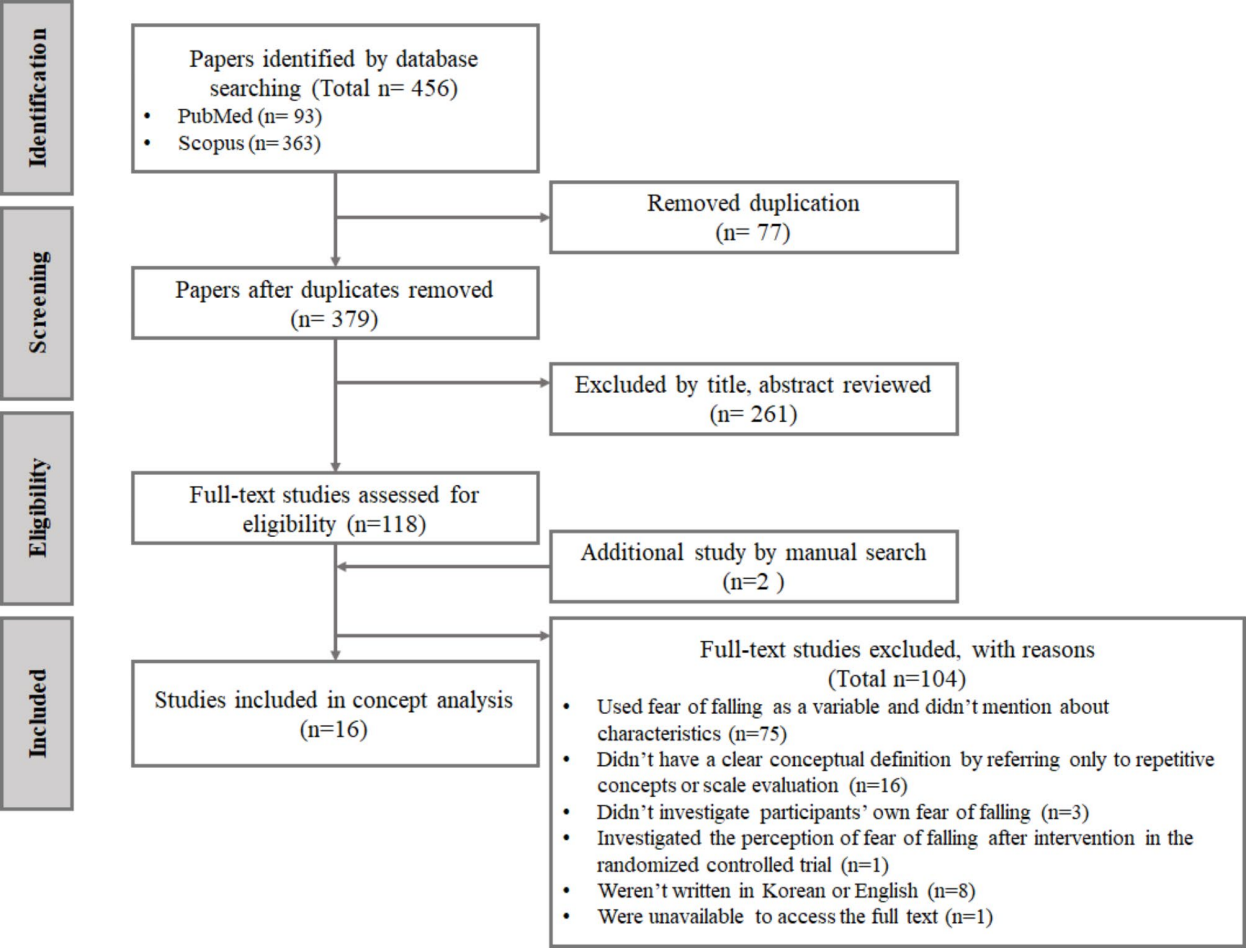
Flow diagram of the study selection

To explore the universal experience of fear of falling in older adults, the literature was included without imposing restrictions based on specific disease conditions. Literature that was not about fear of falling (n = 261), used fear of falling as a variable without mentioning its characteristics (n = 75), didn’t have a clear conceptual definition, and referred only to repetitive concepts or scale evaluation (n = 16), didn’t investigate the participants’ fear of falling (n = 3), investigated the perception of fear of falling after the intervention in the randomized controlled trial (n = 1), weren’t written in Korean or English (n = 8), and were unavailable to access as full text (n = 1) were all removed. A total of 365 documents were removed, and 16 documents were used for the final analysis (Fig. 1).

### Concept analysis process

This study was designed as a concept analysis for clarifying the concept of fear of falling in older adults and the analysis was conducted by Walker & Avant’s eight-step conceptual analysis method [18]. Each step is as follows. (1) Concept selection, (2) Establishing the purpose of concept analysis, (3) Identifying all use of concepts, (4) Identifying the proper attributes of concepts, (5) Presenting a model case of the concept, (6) Presenting a borderline, related, contrary case of the concept, (7) Identifying the antecedents and the consequences, and (8) Presenting the empirical referents.

When deriving the attributes, antecedents, and results of the concept, all the contents mentioned about the fear of falling in the selected literature were identified. Common items were confirmed, and even if they were not found in common, important contents were also reviewed. In particular, during this process, not only the results presented in the selected articles but also the direct words and expressions of the subjects presented by each researcher were verified. After that, the contents revealed in the results of each study were classified into each attribute and collected through a content analysis process [19]. The above process was carried out multiple times, and each time the decisions were made through discussions and agreements between researchers. Then, based on the identified attributes of the fear of falling, three cases were presented, including the model case, a borderline case, and a contrary case. The authors’ clinical and research backgrounds were used to construct these case scenarios.

## Results

### Dictionary definition

The definition of the term “fear of falling” wasn’t found, but “fear” was defined as “the bad feeling that you have when you are in danger or when a particular thing frightens you” and “fall” was explained as “to drop down from a higher level to a lower level” or “to suddenly stop standing” in Oxford dictionary [20, 21]. Also, a fall was defined as “an event which results in a person coming to rest inadvertently on the ground or floor or other lower level” by World Health Organization (WHO) [22]. As such, the meaning of the fear of falling may be derived from the definitions of ‘fall’ and ‘fear’.

### Use of the concept

There are various scales for measuring fear of falling, and each scale defines fear of falling using different concepts. Fear of falling was initially operationalized as a state of ‘low perceived self-efficacy at avoiding falls during essential, non-hazardous activities of daily living’ by Tinetti et al. [16] and was then measured by the Falls Efficacy Scale (FES), which investigates the degree of perceived self-efficacy when performing a particular activity [16]. Additionally, Lachman et al. [23] developed the Survey of Activities and Fear of Falling (SAFFE), which measures fear of falling that doesn’t lead to negative consequences such as activity curtailment or fatality [23]. There is also the Falls Efficacy Scale-International (FES-I), which was developed from FES and includes question items about concerns regarding falling while performing various activities [24, 25]. Lastly, Dayhoff et al. [26] developed the Fear of Falling Questionnaire (FFQ) to measure fear of falling as harm outcomes, coping potential, degree of threat, and future expectancy based on Lazarus’s cognitive appraisal model of emotion [26].

Recently, the term ‘concern about falling’ has emerged, as suggested by The World Falls Guidelines Concern about Falls and Falling Working Group [27, 28]. They recommended using the term ‘concern about falling’ instead of ‘fear of falling’, citing that older adults prefer the term ‘concern’ over ‘fear’ and that ‘concern’ is more suitable and acceptable to older adults because it is perceived as ‘less intense and emotional’ than ‘fear’ [27, 28].

In one previous study presenting a new perspective on falls efficacy, researchers defined falls efficacy as “perceived self-efficacy to perform activities of daily living without falling” and fear of falling as “lasting concerns about falling that lead to individuals avoiding activities they are still capable of performing” [17]. Thus, there has been increasing research that distinguishes fear of falling from falls efficacy.

### Attributes of fear of falling in older adults

In this study, four decisive attributes of fear of falling were presented based on the contents of several previous studies, especially qualitative studies expressing the fear of falling by the participants: Apprehension caused by the unpredictable nature of falls, unease related to own vulnerability, high vigilance-related to the environment, and concern about potential harm after fall events (Table 1).

**Table 1:** Literature included in the concept analysis

No	Author	Year	Title
1	Ellmers TJ, Wilson MR, Norris M, Young WR.	2022	Protective or harmful? A qualitative exploration of older people’s perceptions of worries about falling.
2	Hamed K, Roaldsen K, Halvarsson	2021	“Fear of falling serves as protection and signifies potential danger”: A qualitative study to conceptualise the phrase “fear of falling” in women with osteoporosis.
3	Matsuda PN, Hoffman JM.	2022	Patient perspectives on falls in persons with multiple sclerosis.
4	Thiamwong L, Decker VB.	2020	Overcoming an Irrational Fear of Falling: A Case Study.
5	Yein N, Pal S.	2018	Qualitative study on salient factors influencing Indian elderly’s perception on fall and its related interventions.
6	Dingová M, Králová E.	2017	Fear of falling among community dwelling older adults.
7	Honaker JA, Kretschmer LW.	2014	Impact of fear of falling for patients and caregivers: perceptions before and after participation in vestibular and balance rehabilitation therapy.
8	Jellesmark A, Herling SF, Egerod I, Beyer N.	2012	Fear of falling and changed functional ability following hip fracture among community-dwelling elderly people: an explanatory sequential mixed method study.
9	Kong KSw, Lee Fk, Mackenzie AE, Lee DT.	2002	Psychosocial consequences of falling: the perspective of older Hong Kong Chinese who had experienced recent falls.
10	Jonasson SB, Nilsson MH, Lexell J, Carlsson G.	2018	Experiences of fear of falling in persons with Parkinson’s disease–a qualitative study.
11	Mahler M, Sarvimäki A.	2012	Fear of falling from a daily life perspective; narratives from later life.
12	Lee F, Mackenzie L, James C.	2008	Perceptions of older people living in the community about their fear of falling.
13	Tischler L, Hobson S.	2005	Fear of falling: a qualitative study among community-dwelling older adults.
14	Ward-Griffin C, Hobson S, Melles P, Kloseck M, Vandervoort A, Crilly R.	2004	Falls and fear of falling among community-dwelling seniors: The dynamic tension between exercising precaution and striving for independence.
15	Huang T-T.	2005	Managing fear of falling: Taiwanese elders’ perspective.
16	Schmid AA, Rittman M.	2007	Fear of falling: an emerging issue after stroke.

#### Apprehension caused by the unpredictable nature of falls

It refers to an anxious feeling caused by the unpredictable character of the fall. As a characteristic that affects the occurrence of fear of falling, the concept that the occurrence of falls cannot be predicted has emerged. Due to the nature of falls, the participants thought that they could fall at any time [29], which resulted in increased fear [30]. Fear of falling increased when they were alone because they felt that they couldn’t cope with falls by themselves [31]. Also, the fear of falling tended to decrease when someone was around or accompanied by others, but the attitude of others or the help provided by others sometimes increased the fear of falling [31].

In previous studies, it was said that fear of falling was caused by not being sure of the cause of instability related to falls or thinking that it was out of one’s control [32]. It was reported that these self-uncontrollable instabilities were frequent but unpredictable, and for this reason, they expected themselves to fall every moment they took a step [30, 32]. Also, they thought that they don’t fall every day but the falls could happen suddenly, anytime, anywhere [29, 33]. Along with these characteristics of falls, unpredictable outside characteristics such as weather increased this fear [34, 35].

#### Unease related to own vulnerability

It means one’s vulnerability to falling occurrences and the ability to deal with falls, such as the possibility of fall occurrences, recovery after falls, coping with falls, etc. For individuals, being careful not to fall relates to knowing about their safety and avoiding perceived threats around them [30, 35]. In previous studies, the participant explained that fear of falling meant being careful when taking steps [30]. Fear of falling was associated with discomfort from not being able to fully coordinate themselves and was more frequent when they were tired or stressed [29]. They also expressed concerns about how to get up and recover from fall events [36, 37], and this was related to their perception of their inability to cope with falls.

They said they were conscious of their instability, and expressed that they were not the same as before and they should walk slower now [38]. Also, they thought that they fall because they weren’t paying enough attention, and they said they should be more cautious and careful [34]. They were more conscious of the activities they could perform and places they could visit because of their fear of falling [39] and reported that they strictly planned every day to maintain a good balance [34]. They recognized that their bodies are aging and expressed that this aging process made them more vulnerable to falls [35, 39, 40]. Reduced balance control, decreased vision and hearing, dizziness-related instability, and osteoporosis due to aging were associated with a fear of falling, and the older, lonely, and weaker they were, the stronger their fear of falling became [32, 34, 35, 39, 40]. These feeling of weakness and vulnerability was caused by the lack of trust in their body. The gap between what the brain wants and what the body performs causes fear and anxiety [34].

#### High vigilance related to the Environment

It means paying more attention to the surrounding environment. They explained that fear of falling always makes them more vigilant or careful, and search for hazards that exist in the environment [29].

The design of the surrounding environment played an important role in the fall occurrence, and they expressed that they were highly vigilant to avoid falls and that they need to know better about the surroundings [39]. They explained that they fall because of the physical environment and so, they avoided visiting places with slippery surfaces or stairs [29, 36]. They also argued that they felt unsafe in the crowd, and the fear of falling made the environment look dangerous and overbearing [29, 32]. In previous studies, participants said that if the floor is hard, they feel fear because they think that they will get severely injured when they fall, and if the floor is shiny they think that the floor is slippery, and feel scared even if it is not [29]. In addition, they cannot stand the mess of the environment, especially the floor, and to prevent falls, they organize the surroundings and implement behavioral adjustments such as drying the floor off, to remove risk factors [35].

#### Concern about potential harm after falls

It refers to a wide range of physical, functional, and emotional damage that may occur to an individual after experiencing a fall. Concerns about physical damage in future falls, including injuries, pain, and disabilities had been most often found [29]. In previous studies, many participants thought that falls could result in injuries or fractures, and if they have bad falls, they could injure their heads or break their necks, and become permanently disabled or die [29–38, 41, 42]. They thought of falls as a link to possible physical damage [38] and were concerned about post-fall pain and the possibility that they couldn’t get up again [29]. Some previous studies reported that the fear of falling was a concept developed based on physical damage and loss of function and that the participants experienced fear of possible injury [42].

Also, the participants often mentioned that they were afraid of becoming incompetent, being a burden, or being useless [42], and being incompetent meant a situation in which they had to rely on others for their needs. They expressed fear that they would lose their independence and become dependent on wheelchairs and were worried that they would have to leave their residence and become institutionalized [30, 42].

The persistent fear of falling acted as a psychological pressure on older adults, because of which they became more sensitive, and this heightened sensitivity made them complain about bad moods [35]. In addition, they explained that the fall experience was related to social embarrassment. They were not only embarrassed by the fact that they fell, but also felt humiliated in public when others came and helped them [34, 42]. They reported that they hated being seen and being helped by others, and this affected their confidence [42]. Some of them also mentioned that they heard others laugh when they fell, so they felt uneasy and concerned [34].

### Model case

A 65-year-old woman visited her friend who fell on the street and was hospitalized a few days ago, and she almost fell in the bathroom on the previous day. She felt anxious. After all, she couldn’t predict when the fall will occur and is worried. She thought herself vulnerable to falling because she is old and can’t move as before. In addition, she thinks she would break her leg or be hospitalized if she fell, and she thinks that she should take a closer look at the surroundings when using stairs or doing outdoor activities.

### Borderline case

A 70-year-old woman almost slipped on her way home on a rainy day. She was worried about the thought that she could get seriously injured if she fell. Also, she thought that it was more dangerous because there were many solid objects on the porch. In addition, she was anxious because she thought that a fall could happen to anyone at any time, especially on such a rainy day, so she put an anti-slip mat on the floor.

There is no mention of ‘unease related to own vulnerability’.

### Contrary case

A 75-year-old man almost fell yesterday while using stairs. However, he thought that he wouldn’t fall and that he wouldn’t be seriously injured if he fell. In addition, he thought that if he paid attention, he wouldn’t fall on the stairs and that he should exercise in the future to develop his leg muscles more.

### Antecedents and consequences

Similarities were identified in terms of the antecedents or consequences of fear of falling between the experiences of community-dwelling healthy older adults and patients with various diseases, with the exception of individuals with Parkinson’s Disease. Due to the disease-specific nature of Parkinson’s Disease and the progressive deterioration of their physical condition, their experiences tended to be distinct and specific to the disease.

### Antecedents

**Awareness of Falls and Near Falls.** It means knowing that falls are dangerous. Various opinions were suggested as to whether encountering other people’s fall experiences increases the participants’ fear of falling [29, 30, 43], but it was confirmed that recognizing how dangerous a fall is, including the consequences that may occur after a fall, is the base of the fear of falling. They also expressed that falls could have negative consequences, or that they should be careful to avoid falls [30, 35].

**Direct/Indirect Experience about Falls and Near Falls.** The antecedent factors of fear of falling identified through previous studies were direct and indirect experiences of falls, threats perceived through near-fall experiences where they almost fell but managed to restore balance and changes in the environment [10, 29]. Fear of falling also occurred after experiencing falls or depression or anxiety related to falls, or serious falls requiring medical intervention [30, 36, 40, 42], friends’ or relatives’ experiences of bad falls [29, 36].

### Consequences

Moderate fear of falling has a fall prevention effect, but excessive fear of falling has resulted in increased fall risk, decreased physical activity, decreased social activity, instability, loss of independence, unsafe environment/place/activity avoidance, weakened muscle strength, and poor quality of life [30, 38].

**Protective Effect.** It was said that worrying thoughts have a protective effect because they attract attention to potential risks, which can increase concentration on the task and maintain the correct movements required to maintain safety [32]. In addition, it is said that they are more positive and actively engaged in life so that negative thoughts don’t encroach their life [34, 42], and prepare strategies to reduce the probability of falls, such as improving the home environment or using aids [35, 36].

**Activities Curtailment.** It is reported that fear of falling forces individuals to avoid actions that are thought to perform without falling, thereby reducing the number of safe activities available to participate in, which eventually leads to avoidance and immobility [32]. They avoided activities they enjoyed in the past [39, 42], partially, temporarily, or completely abandoned activities such as using stairs, or taking walks, and restricted themselves to perform only activities they felt confident in [36, 40, 44].

**Reduction in the Radius of Living.** They thought their lives were shrinking. They moved less, and reduced activities in their daily lives [29, 30, 45]. They also described themselves as not leaving the house because they prefer to sit or lie on the sofa, making themselves lazy [41].

**Restricted Freedom.** They described their freedom as limited to living complete and undisturbed lives [34], and said that they would have been free to live more actively in public if there were no fear of falling [29].

**Fig. 2:**
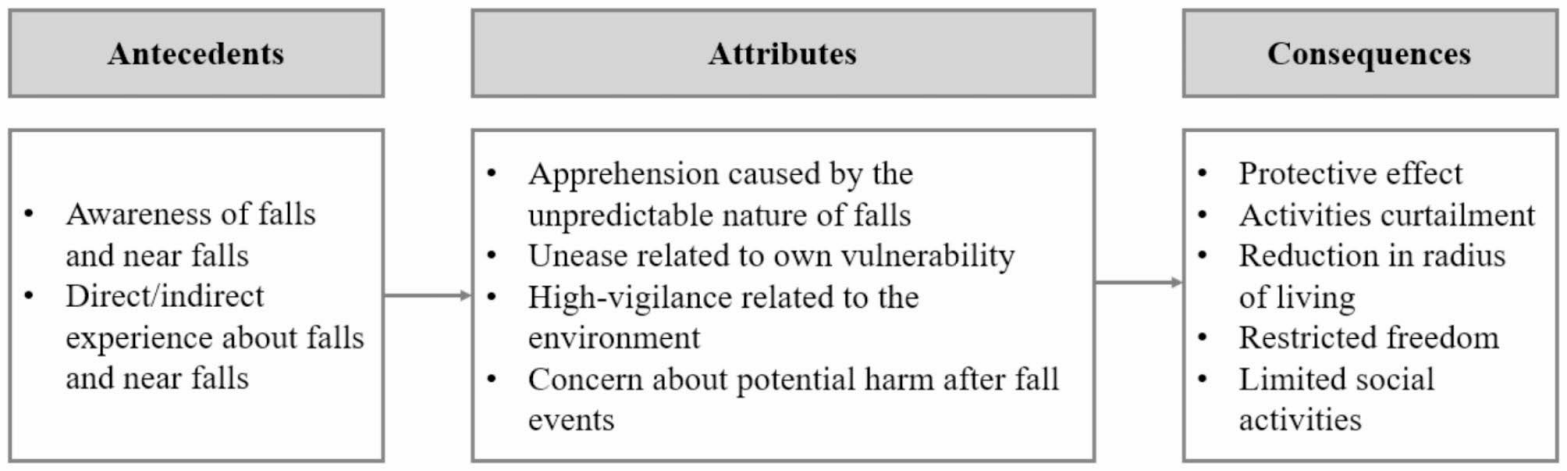
Conceptual Structure of the Fear of Falling in Older Adults

**Limited Social Activities.** They expressed that fear of falling affects socializing with others, and they have difficulty spending time with friends and relatives [34]. It is said that social activities decrease, resulting in social isolation (Fig. 2) [29, 40, 41].

### Empirical referents

Identifying empirical referents is a crucial process for validating the actual manifestation of a concept in the real world [18]. It should be noted that empirical referents refer not only to the measurement tools of the concept but also to the means of assessing its attributes, often aligning with the identified attributes [18]. Thus, in order to achieve this objective, we examined how well the factors comprising the Fear of Falling Questionnaire (FFQ), a tool developed for measuring fear of falling, corresponded to the attributes of fear of falling identified in this study.

In the FFQ developed by Dayhoff et al. [26], the harm outcomes factor measured the anticipated physical and existential harm and fear associated with these anticipations. The statements for measuring this factor included that they would not be able to easily recover from injuries caused by falls, and they would have to stop doing some activities if they fell [26]. Also, in the case of the degree of threat factor, it meant the amount of anxiety caused by the expected threat, and statements measuring this included that their lives would change if they fell [26]. It can be seen that these are in line with the ‘concern about potential harm after fall events’ of this study. Another factor of FFQ, coping potential, referred to an evaluation of an individual’s ability to cope with a change, and as a measure of this, statements that they believe that they would not fall, and they might make a change not to fall included [26]. This is related to the ‘unease related to own vulnerability’ and ‘high vigilance-related to the environment,’ mentioned in this study. However, it was difficult to find factors or items related to ‘apprehension caused by the unpredictable nature of falls.’

## Discussion

As discussed in this study, four attributes make up the fear of falling: apprehension caused by the unpredictable nature of falls, unease related to own vulnerability, high vigilance related to the environment, and concern about potential harm after fall events. There are two antecedents of fear of falling which are awareness of falls and near falls, and direct/indirect experience about falls and near falls, and five consequences of fear of falling which are protective effect, activities curtailment, reduction in radius of living, restricted freedom, and limited social activities. This is in line with the cognitive vulnerability model of the etiology of fear proposed by Armfield [46]. Armfield [46] suggested the vulnerability schema as an important factor in the outbreak of fear [46]. It was proposed that this vulnerability schema included the uncontrollability and unpredictability of fear stimuli, and was also associated with the possibility that these fear stimuli can lead to negative consequences which are related to danger and disgust [46]. Also, the vulnerability schema was associated with learning experiences related to the fear stimuli or individual characteristics [46]. After activation of the vulnerability schema, two processes induced fear: the first is to elicit immediate fear with an automatic response, and the second is to include the relatively slow, automatic, semi-automatic, and non-automatic aspects, leading to cognitive fear along with the evaluation process [46]. It was stated that this overall process could lead to emotional, physiological, and behavioral reactions, and the interaction of these results goes back and influence the vulnerability schema [46].

Among the results of this study, ‘apprehension caused by the unpredictable nature of falls’ which is one of the attributes of fear of falling is said to be associated with unpredictability among the vulnerability schema. In previous studies, unpredictability was related to Spatio-temporal unpredictability, perceived risk, and viability [46, 47], and when looking at animal-related fears as examples, the inability to predict animals’ movement, encounters, or willingness to attack was included in the unpredictability [46, 48]. Therefore, if this is applied to falls, it is related to the aspect of uncertainty which includes that it is hard to know when falls will occur. Since this attribute to psychological anxiety occurs with the uncertainty and unpredictability of fall events, it is related to the unpredictability of this theory. A prior study mentioned that the fear of falling increases because people were unable to anticipate their next fall. This finding aligns with the unpredictability emphasized in theory [28].

Subsequently, ‘concern about potential harm after fall events’ seems to be related to unpredictability, danger, and disgust among vulnerability schema. One of the properties of unpredictability presented in the literature is the ‘unpredictability of suffering actual harm if attacked’ [46]. If this aspect is put into a situation related to falls, it can be said that it is impossible to know how many injury outbreaks when a fall occurs. In addition, participants reported that they were worried about various physical and functional difficulties that could occur after fall events, which can be seen as reflecting the aspect of danger among vulnerability schema because it is related to the risk of falling. Also, one of the injuries that participants were worried about was emotional damage, which they expressed as the social embarrassment they would experience as they fall, and expressed that they would be humiliated [34, 42]. This aspect seems to reflect the disgust among vulnerability schema.

In the previous studies, the perceived vulnerability was explained to be an uncomfortable emotion related to capacity or an inability related to stimuli, to which an unexpected and unaffordable shock had a detrimental effect [46, 49]. The attribute ‘unease related to own vulnerability’ identified in this study could also be related to this aspect, because the participants reported that they thought they were susceptible to falls or they were easily injured when exposed to falls. This aspect could be related to the possibility of participants encountering more frequent falls or greater risk concerning the attribute of danger among the vulnerability schema.

Lastly, ‘high vigilance related to the environment’ can be associated with the notion of uncontrollability in the vulnerability schema. A prior study suggested that when explaining uncontrollability in relation to fear of certain animals, one factor is the inability to control encounters with the animal or to avoid them altogether [46]. If we apply this perspective to falls, it includes scenarios where the occurrence of falls cannot be managed, and the start and end of a fall cannot be controlled. This attribute elucidates that vigilance towards the hazardous environment increases due to the uncontrollable nature of falls, which establishes a connection to the concept of uncontrollability in falls. Moreover, this component, previously identified as “feeling out of control” and “lack of perceived control”, supports the notion of uncontrollability in this theory [31].

Furthermore, personality factors and learning experiences were said to be factors influencing the vulnerability schema in this theory [46]. Similarly, ‘awareness of falls and near falls’, and ‘direct/indirect experience about falls and near falls’ were suggested as antecedents of the fear of falling in this study, which could be seen as learning experiences of this theory. It was suggested that various reactions could occur due to the progress of the activated vulnerability schema, and similarly, this study confirmed that psychological, behavioral, and social reactions appeared as a result of fear of falling.

Although this study was not conducted based on this theory, there was a correspondence when considering each attribute and concept in this theory. Through this, it can be seen that the results of this study reflect the overall aspect of fear occurrence, while also encompassing the characteristics related to falls.

In the previous study, falls efficacy was identified as corresponding to the cognitive domain, while fear of falling was associated with the emotional domain [17]. This indicates that all attributes related to various aspects of emotion are important components of fear of falling. Specifically, four attributes can be distinguished based on the causes of fear, which include characteristics of fall occurrences, the subject’s vulnerability, environmental risks, and the outcomes of falls. Various triggers can evoke the emotion of fear. Among these attributes, two that stand out as differentiating fear of falling from falls efficacy are ‘appreciation caused by the unpredictable nature of falls’ and ‘concern about potential harm after falls’.

‘Appreciation caused by the unpredictable nature of falls’ is characterized by the realization that a fall can happen suddenly anywhere, irrespective of the subject’s abilities or confidence. This awareness of the potential risk of falling evokes strong emotions in individuals. Additionally, ‘concern about potential harm after falls’ generates fear due to the possibility of various damages that may occur following a fall. It is distinctly different from confidence or efficacy in preventing falls. Notably, it can be seen as an attribute that sets fear of falling apart from falls efficacy, as the perception of falling risk by an individual directly contributes to the experience of fear.

Overall, fear of falling is a complex concept that encompasses various environmental and situational factors in addition to individual factors. This unique characteristic distinguishes it from other concepts.

### Limitation

Most of the studies included in the analysis investigated the fear of falling in older adults, but some studies included participants under the age of 65 with more focus on specific diseases such as stroke, Parkinson’s disease, and dizziness. However, many studies focused on older adults, and certain disease-specific factors revealed in some studies were excluded because they weren’t commonly revealed in several studies, so it was unlikely to have affected the overall characteristics of fear of falling in older adults.

In addition, there is a limitation in that the number of documents used in the analysis isn’t large. However, all literature has the advantage of being able to confirm deep and diverse opinions on the concept of fear of falling by using qualitative approaches, which seems to compensate for the limitation.

### Implications

Understanding the antecedents, attributes, and consequences of fear of falling is crucial for clinicians when developing strategies for fall prevention in older adults. Based on the study’s findings, the fear of falling is rooted in individual recognition of the potential danger posed by a fall. Therefore, clinicians should gather data on direct and indirect experiences of falls, as these experiences can influence the degree of fear.

The findings reveal that fear of falling in older adults manifest in four distinct aspects: apprehension arising from the unpredictability of falls, unease related to personal vulnerability, heightened vigilance concerning the environment, and concern about potential harm following fall events. The extent to which individuals experience each aspect may differ. Consequently, interventions should be tailored to address these aspects on a personalized level. Implementing effective fall prevention programs can assist in reducing apprehension, enhancing perceived control, promoting physical strength through exercise, addressing emotional vulnerability, improving environmental safety, and increasing knowledge about falls.

Considering that an excessive fear of falling can result in activity restriction, limited mobility, reduced independence, and decreased social engagement, it is imperative to evaluate the nature and scope of fear of falling in order to enhance the overall quality of life for older adults. Fall prevention programs should incorporate these aspects of fear of falling, particularly by providing education and counseling interventions to older adults experiencing the negative consequences of excessive fear. Additionally, public awareness campaigns and policy implementation are necessary to foster safe indoor and outdoor environments for older adults.

## Conclusion

In this study, to understand the fear of falling, which is important for older adults, the attributes, antecedents, and consequences of this concept were explored. It was confirmed that the unique characteristics of falls and the fear-inducing process were fused to form the fear of falling. Based on the identified attributes, the fear of falling is an emotional state characterized by apprehension stemming from the unpredictable nature of falls, coupled with unease regarding one’s vulnerability. It entails a state of high vigilance towards the environment and is accompanied by significant concern about the potential harm that may occur following fall events. Thus, the fear of falling involves not only emotional responses (apprehension, unease, concern) but also cognitive response (vigilance) to a perceived threat. This is believed to be the basis for developing scales that measure various aspects of fear of falling in the elderly in future studies. In addition, it is possible to present what aspects should be identified and considered when identifying or intervening in the fear of falling, which is expected to be an important basis for future research on the fear of falling or for dealing with the fear of falling in the clinical field.

## Data Availability

All data generated or analysed during this study are included in this published article.

## References

[1] Cruz-Díaz D, Martínez-Amat A, Manuel J, Casuso RA, de Guevara NML, Hita-Contreras F (2015). Effects of a six-week pilates intervention on balance and fear of falling in women aged over 65 with chronic low-back pain: a randomized controlled trial. Maturitas.

[2] Sapmaz M, Mujdeci B (2021). The effect of fear of falling on balance and dual task performance in the elderly. Exp Gerontol.

[3] Scholz M, Haase R, Trentzsch K, Weidemann ML, Ziemssen T (2021). Fear of falling and falls in people with multiple sclerosis: a literature review. Multiple Scler Relat Disorders.

[4] Liu M, Hou T, Li Y, Sun X, Szanton SL, Clemson L (2021). Fear of falling is as important as multiple previous falls in terms of limiting daily activities: a longitudinal study. BMC Geriatr.

[5] Hoang OTT, Jullamate P, Piphatvanitcha N, Rosenberg E (2017). Factors related to fear of falling among community-dwelling older adults. J Clin Nurs.

[6] Delbaere K, Close JC, Brodaty H, Sachdev P, Lord SR. Determinants of disparities between perceived and physiological risk of falling among elderly people: cohort study. BMJ. 2010;341. 10.1136/bmj.c4165 PMC2930273 20724399

[7] Mazumder R, Lambert WE, Nguyen T, Bourdette DN, Cameron MH (2015). Fear of falling is associated with recurrent falls in people with multiple sclerosis: a longitudinal cohort study. Int J MS care.

[8] Pereira C, Bravo J, Raimundo A, Tomas-Carus P, Mendes F, Baptista F (2020). Risk for physical dependence in community‐dwelling older adults: the role of fear of falling, falls and fall‐related injuries. Int J Older People Nurs.

[9] Scheffer AC, Schuurmans MJ, Van Dijk N, Van Der Hooft T, De Rooij SE (2008). Fear of falling: measurement strategy, prevalence, risk factors and consequences among older persons. Age Ageing.

[10] Hadjistavropoulos T, Delbaere K, Fitzgerald TD (2011). Reconceptualizing the role of fear of falling and balance confidence in fall risk. J Aging Health.

[11] Friedman SM, Munoz B, West SK, Rubin GS, Fried LP (2002). Falls and fear of falling: which comes first? A longitudinal prediction model suggests strategies for primary and secondary prevention. J Am Geriatr Soc.

[12] Weijer RH, Hoozemans MJ, Meijer OG, van Dieën JH, Pijnappels M (2021). The short-and long-term temporal relation between falls and concern about falling in older adults without a recent history of falling. PLoS ONE.

[13] Lavedán A, Viladrosa M, Jürschik P, Botigué T, Nuín C, Masot O (2018). Fear of falling in community-dwelling older adults: a cause of falls, a consequence, or both?. PLoS ONE.

[14] Litwin H, Erlich B, Dunsky A (2018). The complex association between fear of falling and mobility limitation in relation to late-life falls: a SHARE-based analysis. J Aging Health.

[15] Moore DS, Ellis R (2008). Measurement of fall-related psychological constructs among independent-living older adults: a review of the research literature. Aging and Mental Health.

[16] Tinetti ME, Richman D, Powell L (1990). Falls efficacy as a measure of fear of falling. J Gerontol.

[17] Soh SL-H, Tan C-W, Thomas JI, Tan G, Xu T, Ng YL (2021). Falls efficacy: extending the understanding of self-efficacy in older adults towards managing falls. J Frailty Sarcopenia Falls.

[18] Walker LO, Avant KC (2019). Strategies for theory construction in nursing.

[19] Cole FL (1988). Content analysis: process and application. Clin Nurse Specialist.

[20] Press OU. fear [Available from: https://www.oxfordlearnersdictionaries.com/definition/english/fear_1?q=fear.

[21] Press OU. fall [Available from: https://www.oxfordlearnersdictionaries.com/definition/english/fall_1?q=fall.

[22] Organization WH. Falls 2021 [updated 2021.04.26. Available from: https://www.who.int/news-room/fact-sheets/detail/falls.

[23] Lachman ME, Howland J, Tennstedt S, Jette A, Assmann S, Peterson EW (1998). Fear of falling and activity restriction: the survey of activities and fear of falling in the elderly (SAFE). The Journals of Gerontology Series B: Psychological Sciences and Social Sciences.

[24] Bower ES, Wetherell JL, Merz CC, Petkus AJ, Malcarne VL, Lenze EJ (2015). A new measure of fear of falling: psychometric properties of the fear of falling questionnaire revised (FFQ-R). Int Psychogeriatr.

[25] Yardley L, Beyer N, Hauer K, Kempen G, Piot-Ziegler C, Todd C (2005). Development and initial validation of the Falls Efficacy Scale-International (FES-I). Age Ageing.

[26] Dayhoff N, Baird C, Bennett S, Backer J (1994). Fear of falling: measuring fear and appraisals of potential harm. Rehabilitation Nurs Res.

[27] Ellmers TJ, Freiberger E, Hauer K, Hogan DB, McGarrigle L, Lim ML (2023). Why should clinical practitioners ask about their patients’ concerns about falling?. Age Ageing.

[28] Montero-Odasso M, van der Velde N, Martin FC, Petrovic M, Tan MP, Ryg J (2022). World guidelines for falls prevention and management for older adults: a global initiative. Age Ageing.

[29] Jonasson SB, Nilsson MH, Lexell J, Carlsson G (2018). Experiences of fear of falling in persons with Parkinson’s disease–a qualitative study. BMC Geriatr.

[30] Lee F, Mackenzie L, James C (2008). Perceptions of older people living in the community about their fear of falling. Disabil Rehabil.

[31] Ward-Griffin C, Hobson S, Melles P, Kloseck M, Vandervoort A, Crilly R (2004). Falls and fear of falling among community-dwelling seniors: the dynamic tension between exercising precaution and striving for independence. Can J Aging/La Revue Canadienne du Vieillissement.

[32] Ellmers TJ, Wilson MR, Norris M, Young WR (2022). Protective or harmful? A qualitative exploration of older people’s perceptions of worries about falling. Age Ageing.

[33] Mahler M, Sarvimäki A (2012). Fear of falling from a daily life perspective; narratives from later life. Scand J Caring Sci.

[34] Hamed K, Roaldsen K, Halvarsson A (2021). Fear of falling serves as protection and signifies potential danger: a qualitative study to conceptualise the phrase fear of falling in women with osteoporosis. Osteoporos Int.

[35] Huang T-T (2005). Managing fear of falling: taiwanese elders’ perspective. Int J Nurs Stud.

[36] Dingová M, Králová E (2017). Fear of falling among community dwelling older adults. Cent Eur J Nurs Midwifery.

[37] Kong KSw L, Fk, Mackenzie AE, Lee DT (2002). Psychosocial consequences of falling: the perspective of older Hong Kong Chinese who had experienced recent falls. J Adv Nurs.

[38] Schmid AA, Rittman M (2007). Fear of falling: an emerging issue after stroke. Top Stroke Rehabil.

[39] Matsuda PN, Hoffman JM (2022). Patient perspectives on falls in persons with multiple sclerosis. PM&R.

[40] Thiamwong L, Decker VB (2020). Overcoming an Irrational fear of falling: a Case Study. Clin Case Stud.

[41] Jellesmark A, Herling SF, Egerod I, Beyer N (2012). Fear of falling and changed functional ability following hip fracture among community-dwelling elderly people: an explanatory sequential mixed method study. Disabil Rehabil.

[42] Tischler L, Hobson S (2005). Fear of falling: a qualitative study among community-dwelling older adults. Phys Occup Therapy Geriatr.

[43] Howland J, Lachman ME, Peterson EW, Cote J, Kasten L, Jette A (1998). Covariates of fear of falling and associated activity curtailment. Gerontologist.

[44] Yein N, Pal S, editors. Qualitative study on salient factors influencing Indian elderly’s perception on fall and its related interventions. Advances in Design for Inclusion: Proceedings of the AHFE 2017 International Conference on Design for Inclusion, July 17–21, 2017, The Westin Bonaventure Hotel, Los Angeles, California, USA 8; 2018: Springer.

[45] Honaker JA, Kretschmer LW. Impact of fear of falling for patients and caregivers: perceptions before and after participation in vestibular and balance rehabilitation therapy. 2014. 10.1044/1059-0889(2013/12-0074) 23824441

[46] Armfield JM (2006). Cognitive vulnerability: a model of the etiology of fear. Clin Psychol Rev.

[47] Merckelbach H, Van den Hout MA, Jansen A, van der Molen GM (1988). Many stimuli are frightening, but some are more frightening than others: the contributions of preparedness, dangerousness, and unpredictability to making a stimulus fearful. J Psychopathol Behav Assess.

[48] Arntz A, Van Eck M, de Jong P (1991). Avoidance of pain of unpredictable intensity. Behav Res Ther.

[49] Weiss JM (1971). Effects of coping behavior in different warning signal conditions on stress pathology in rats. J Comp Physiological Psychol.

